# High-throughput m^6^A-seq reveals RNA m^6^A methylation patterns in the chloroplast and mitochondria transcriptomes of *Arabidopsis thaliana*

**DOI:** 10.1371/journal.pone.0185612

**Published:** 2017-11-13

**Authors:** Zegang Wang, Kai Tang, Dayong Zhang, Yizhen Wan, Yan Wen, Quanyou Lu, Lei Wang

**Affiliations:** 1 College of Bioscience and Biotechnology, Yangzhou University, Yangzhou, China; 2 School of Biotechnology, Jiangsu University of Science and Technology, Zhenjiang, China; 3 Department of Horticulture and Landscape Architecture, Purdue University, West Lafayette, Indiana, United States of America; 4 Institute of Biotechnology, Jiangsu Academy of Agricultural Sciences, Nanjing, China; 5 Sericultural Research Institute, Chinese Academy of Agricultural Sciences, Zhenjiang, China; John Curtin School of Medical Research, AUSTRALIA

## Abstract

This study is the first to comprehensively characterize m^6^A patterns in the *Arabidopsis* chloroplast and mitochondria transcriptomes based on our open accessible data deposited in NCBI's Gene Expression Omnibus with GEO Series accession number of GSE72706. Over 86% of the transcripts were methylated by m^6^A in the two organelles. Over 550 and 350 m^6^A sites were mapped, with ~5.6 to ~5.8 and ~4.6 to ~4.9 m^6^A sites per transcript, to the chloroplast and mitochondria genome, respectively. The overall m^6^A methylation extent in the two organelles was greatly higher than that in the nucleus. The m^6^A motif sequences in the transcriptome of two organelles were similar to the nuclear motifs, suggesting that selection of the m^6^A motifs for RNA methylation was conserved between the nucleus and organelle transcriptomes. The m^6^A patterns of rRNAs and tRNAs in the organelle were similar to those in the nucleus. However, the m^6^A patterns in coding RNAs were distinct between the nucleus and the organelle, suggesting that that regulation of the m^6^A methylation patterns may be different between the nuclei and the organelles. The extensively methylated transcripts in the two organelles were mainly associated with rRNA, ribosomal proteins, photosystem reaction proteins, tRNA, NADH dehydrogenase and redox. On average, 64% and 79% of the transcripts in the two organelles showed differential m^6^A methylation across three organs of the leaves, flowers and roots. The m^6^A methylation extent in the chloroplast was higher than that in the mitochondria. This study provides deep insights into the m^6^A methylation topology and differentiation in the plant organelle transcriptomes.

## Introduction

Chemical modifications have been found ubiquitously distributing in RNAs of the living species[1–10]. Among those, *N*^6^-methyladenosine (m^6^A) has been found prevalently distributing in nuclear mRNA, rRNA, tRNA, and some snRNA of eukaryotes[3,5,7,10–16]. The m^6^A topology was found highly conserved in the eukaryote transcritptome[17,18]. For example, most m^6^A sites enriched near the stop codon or 3'untranslated regions (UTR) in the nuclear mRNAs of the higher living species[17–20]. RNA m^6^A modification in the nuclear RNAs was responsible for certain important metabolisms, e.g. RNA splicing, RNA export[21], RNA stability[17,18], control of the circadian clock[22], regulation of gene expression[23,24], decision of cell differential fate[10,15,25] and regulation of RNA-protein interaction[26]. Silencing the m^6^A methyltransferase significantly influences gene expression and alternative splicing patterns, resulting in initiation of the *p53*signaling pathway and apoptosis [17]. m^6^A modification is selectively recognized by binding proteins to affect the translation status and lifetime of mRNA [18]. Specific inhibition of m^6^A methylation by silencing of the m^6^A methylase *Mettl3* is sufficient to elicit circadian period elongation and RNA processing delay [22]. Increased m^6^A methylation promotes there programming of mouse embryonic fibroblasts(MEFs) to pluripotent stem cells; in contrast, a reduced m^6^A level impedes reprogramming [25]. The methylation and demethylation of m^6^A is contemporarily and precisely regulated to be balanced due to stimuli as to maintain an appropriate metabolism in the cell[24]. Defect in m^6^A methylation or demethylation will result in severe physiological consequences[27], e.g. abnormal reproductive development[28,29], obesity[30], or cancer[10–15,31,32] in mammals.

Most of the mysteries concerning m^6^A RNA methylation were derived from mammals aforementioned. However, some phenomena associated with m^6^A RNA methylation were discovered in plants, which adds our knowledge in this area. Plant mRNA contains m^6^A methylation level similar to that in animal cells[28,33,34]. *N*^6^-methyladenosine mRNA methylase is essential for embryonic development in *Arabidopsis thaliana*[28]. Inactivation of the *Arabidopsis* mRNA adenosine methylase (MTA) results in failure of the developing embryo to arrest at the globular stage [28]. mRNAs in the arrested seeds contain deficient m^6^A methylation [28]. A 90% reduction of m^6^A levels during later growth stages gives rise to plants with altered growth patterns and reduced apical dominance [19]. The flowers of the mutant plants show defects in their floral organ number, size, and identity [19]. MTA expression is highly associated with dividing tissues, particularly reproductive organs, shoot meristems, and emerging lateral roots [28]. Over 85% of the modified transcripts show high m^6^A methylation extent compared to their transcript level in *Arabidopsis thaliana* [35]. Highly m^6^A methylated transcripts are mainly associated with transporters, stress responses, redox, regulation factors, and some non-coding RNAs [35]. m^6^A may be another important contributor to organ differentiation in *Arabidopsis* and rice [34,35]. Most of the transposable element transcripts retain a fragmented form with a relatively low transcript level and high m^6^A methylation in the cells, which is suitable for roles of the transposable elements[35]. Therefore, m^6^A RNA methylation also plays important roles connecting critical metabolisms in plants.

High efficiency and specific binding ability of the m^6^A antibody provides a useful tool for a transcriptome-wide analysis of the m^6^A patterns in several species [17,18,21,30,33,34]. The successful experiments are fulfilled through use of RNA immunoprecipitation (RIP) and m^6^A-seq [17,18,21,30,33,34]. RIP experiment in the m^6^A-seq study was aimed to enrich RNAs containing m^6^A through use of m^6^A antibody to the fragmented RNA pool [17]. The enriched m^6^A RNA pool is used for RNA-seq, called ‘m^6^A-seq’[17,18,21,30,33,34].

Chloroplast and mitochondria are two important organelles mainly for photosynthesis and respiration in plants, respectively[36,37]. Amyloplast was derived from chloroplast and evolved mainly for food storage in plant organs, e.g, in roots, fruits and seeds[38]. m^6^A modification was recently found in the chloroplast transcritptome[34]. But information of m^6^A modification in mitochondria is unclear. In addition, m^6^A methylation differences of RNAs between organelles and nucleus have not been well assayed. The nuclear m^6^A RNA methylation was deeply surveyed in previous studies [17,18,21,30,33,34]. However, little is known of this modification in the organelles. This study aimed to (i) comprehensively characterize m^6^A distributing patterns in the *Arabidopsis* chloroplast/amyloplast and mitochondria transcriptomes, (ii) analyze relationship between the transcript level and the m^6^A modification extent in the *Arabidopsis* chloroplast/amyloplast and mitochondria, and (iii) characterize differential patterns of the m^6^A methylation across leaves, flowers and roots in the *Arabidopsis* chloroplast/amyloplast and mitochondria. This is the first study to comprehensively analyze the m^6^A distributing and differential patterns across organs in the plant organelles.

## Methods

### Ethics statement

All plant materials used in this study are freely available to all researchers without any protection for the intellectual property right. This research meets all applicable requirements for the ethics of experimentation and research integrity from all five institutes that provide support to this study.

### Plant growth conditions and treatments

Wild Columbia ecotype (‘Col-0’) of *Arabidopsis thaliana* was used in this research as we in our recent publication [35]. When the plants were fully flowered (five weeks after seed germination), the materials of flowers, rosette leaves and roots were separately collected, promptly frozen in liquid nitrogen and stored at -80°C until use.

### RNA extraction

The modified cetyl trimethylammonium bromide (CTAB) method was used for RNA isolation as described in our previous study[35]. LiCl solution (8.0 M) was used to precipitate RNA. The purified RNA pellet was stored at -80°C until use.

### RNA fragmentation and RNA immunoprecipitation (RIP)

The purified total RNA was fragmented into ~100-nucleotide-long usingthe ZnCl_2_ buffer (10mM ZnCl_2_ and 10mM Tris-HCl, pH 7) according to the protocol developed by Dominissini et al. [17]. The fragmented RNA was prepared for RIP and m^6^A-seq.

The m^6^A-specific binding antibody (Merck Millipore, Billerica, MA, USA) was used for the RIP experiments according to the protocol of Dominissini et al.’s [17]. Ethanol and glycogen were used to precipitate the pulled-down RNA by the m^6^A antibody. The m^6^A RNA pellet was cleaned using 80% ethanol and then resuspended into 15 μl dd-H_2_O for m^6^A-seq, high-performance liquid chromatography (HPLC) and mass spectrometry (MS) analysis.

### RNA-seq, m^6^A-seq and input RNA-seq

To perform RNA sequencing from numerous RNAs including coding RNAs without polyA in the organelles, the Ribo-Zero rRNA Removal Kit (Madison, WI, USA) was used to remove rRNA (actually rRNA can not be completely removed). High throughput m^6^A-seq, RNA-seq and input RNA-seq were performed on HiSeq 2000 (Illumina Inc, San Diego, CA) at Purdue University Genomics Core Facility (http://www.genomics.purdue.edu/services/core.shtml). All RNA sequencing of three samples of leaves, flowers and roots was performed at the same batch on the same sequencer.

### Alignment of reads and visualization of m^6^A peaks

All RNA sequencing data sets were mapped to the *Arabidopsis* genome (TAIR10) using TopHat2 under a parameter of ‘-b2-fast’[39]. The potential PCR duplicates were removed by a parameter of ‘rmdup’ rooted in SAM tools [40]. The fragment numbers for each transcript were estimated using the feature Counts with a parameter of “-p”[41].

The distributing patterns of the mapped m^6^A-seq data were visualized using free software, Integrative Genomics Viewer (IGV2.3, Boston, MA, USA) [42]. IGV is used for high-performance visualization of interactive exploration of large, integrated genomic datasets [42]. The tool is able to present extent of m^6^A methylation, sequencing depth, sequencing fragment alignment, and gene ID of the sequencing data [42].

Because of an extensively low non-specific immunoprecipitation rate (< 1%) in this study[35], all the mapped reads in the m^6^A-seq were assumed to be derived from specific immunoprecipitation of the RNA fragments containing m^6^A modification. Thus, an estimation of m^6^A peak number of a m^6^A modified transcript was estimated by this formula: total mapped length covered by m^6^A fragments within the transcript/150, considered that library construction for m^6^A-seq was created from a m^6^A RNA pool with an average RNA length of ~106 nucleotides (S1 Fig) and average coverage of a peak in m^6^A-seq data was ~150-nucleotide long in this study as visualized by IGV 2.3 [35].

### Discernment of m^6^A topological patterns

The consensus m^6^A motif sequences were figured out by Luo et al.’s protocol [33]. The typical m^6^A patterns of different types of RNAs were captured by screenshot from IGV2.3 visualization of the m^6^A mapping results after normalization.

### m^6^A methylation extent versus transcript level

Sequencing depth in RNA-seq was normalized using the algorithm of Fragments Per Kilobase of Transcript Per Million Fragments Mapped (FPKM = Counts of mapped fragments × 10^9^) / (Length of transcript × Total count of the mapped fragments))[43]. While the sequencing depth in m^6^A-seq was normalized using a modified FPKM (MFPKM = Counts of mapped fragments × 10^9^) / (Total mapped length covered by m^6^A fragments within the transcript × Total count of the mapped m^6^A fragments))[35].

The m^6^A methylation extent of a transcript were categorized into three groupings based on comparison of MFPKM of the transcript in the m^6^A-seq with FPKM of the same transcript in the RNA-seq using χ^2^ test:(1) the m^6^A methylation extent ‘equivalent’ to the transcript level (‘equivalent’, ratio of FPKM to MFPKM fits 1:1 (*p* < 0.05)), (2) the methylation extent higher than the transcript level (‘Hi’, ratio of FPKM to MFPKM < 1 (*p* < 0.05)), and (3) the methylation extent lower than the transcript level (‘Low’, ratio of FPKM to MFPKM > 1 (*p* < 0.05))[35].

### Differential transcript level and differential m^6^A methylation among plant organs

RNA-seq data was normalized by FPKM as described above. χ^2^ tests were used to estimate whether FPKM was significantly different between two organs using R 3.1 (http://cran.r-project.org/bin/windows/base/). The transcripts with a fold change in FPKM > 2.0 or < 0.5, and FDR < 0.02 were considered differentially expressed between two organs[17].

To minimize influence of the transcript level on estimation of differentiation of the m^6^A extent, m^6^A-seq data was normalized by a specific algorithm, NFPKM (NFPKM = MFPKM in m^6^A-seq/LOG (FPKM in RNA-seq, 2)). χ^2^ tests were also used to estimate whether NFPKM of a m^6^A modified transcript was significantly different between two organs using R 3.1. The transcripts with a fold change of NFPKM > 2.0 or < 0.5, and FDR < 0.005 were considered differentially methylated between two organs[17,35].

### qRT-PCR

**Fig 1:**
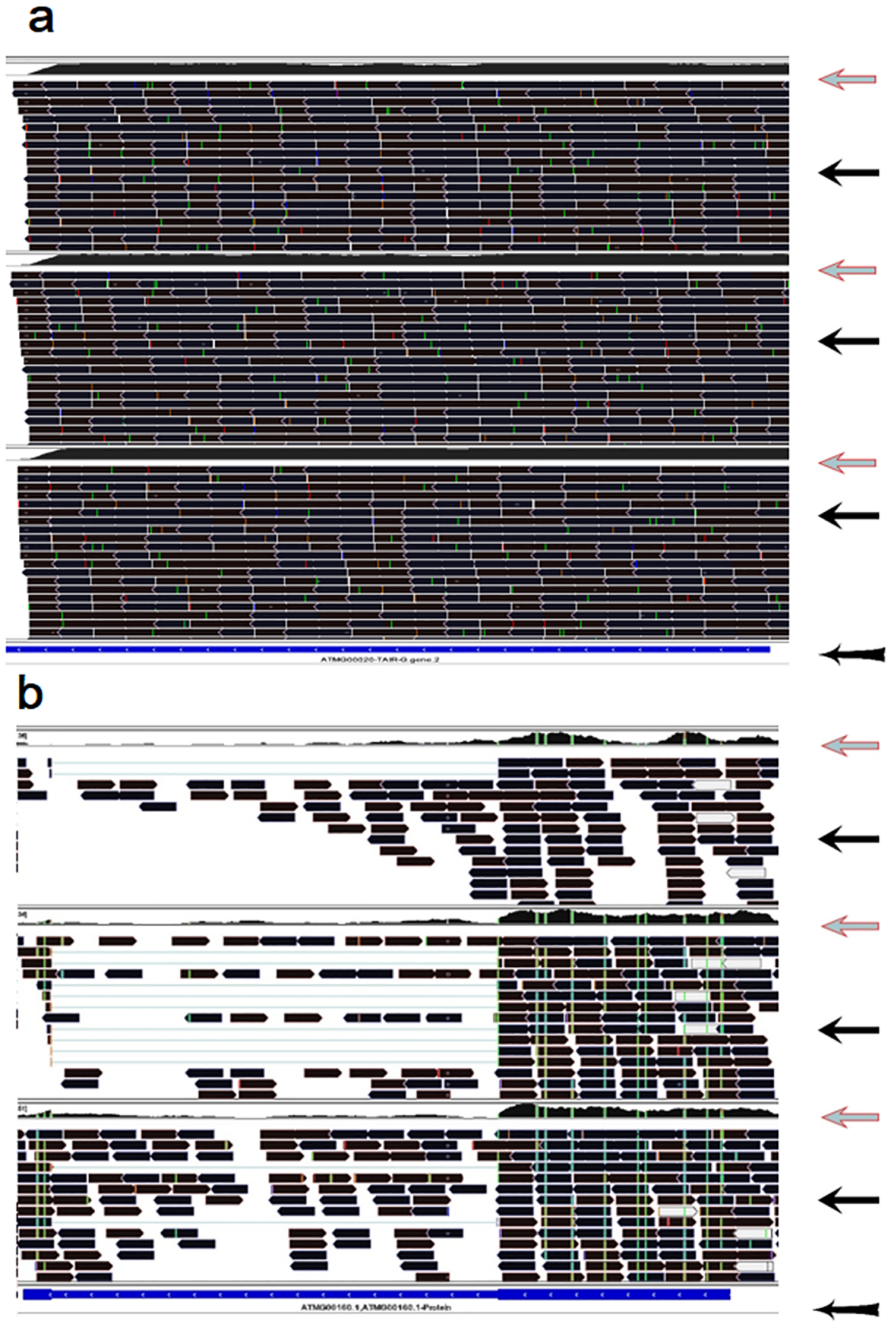
Screenshots from the IGV visualized program present two typical types of m^6^A topologies in the coding RNAs in the *Arabidopsis* mitochondria. Extent of m^6^A methylation, sequencing depth, sequencing fragment alignment, and gene ID of the sequencing data can be clearly visualized by the IGV program [42]. The area in the screenshot indicated by the arrow, “Red leftwards arrow”, presents m^6^A methylation extent across the transcript. The area in the screenshot indicated by the arrow, “Black leftwards arrow”, presents sequencing fragment alignment across the transcript. The area in the screenshot indicated by the arrow, “Black leftwards arrow with tail”, presents gene ID information including gene ID, sequence reading direction, the intron and exon regions. (a) Type 1 (representative gene, ‘ATMG00920’, expressed for ‘a hypothetical protein’), the whole transcript without intron was highly methylated by m^6^A; (b) Type 2 (representative gene, ‘ATMG00160’, expressed for ‘cytochrome oxidase 2’), the exon was highly methylated but the intron was not methylated by m^6^A. Trace files of three organs, leaves (the upper), flowers (in the middle) and roots (the lower) were presented within a screenshot.

Quantitative real-time PCR (qRT–PCR) was performed to assess relative abundance (RA)of m^6^A RNA in the RIP samples. All purified RNA templates were transferred into cDNA using Quanta qScript^™^ cDNA Synthesis Kits (Quanta BioSciences, Inc, Gaithersburg, MD, USA). Six genes were randomly chosen for this test (S1 Table). qRT-PCR primers were designed to span exon-exon junctions in order to eliminate potential amplification of the genomic DNA. qRT-PCR was performed on C1000 Thermal Cycler (Bio-RAD) using SYBR Green SuperMix buffer (Bio-RAD) and 300 ng total cDNA template for amplification. Because the qRT-PCR amplicon spanned an exon-exon junction with a length of 80–150 bp (Fig 1a), cDNA of the *Actin2* gene was used for housekeeping gene and was used for normalization of total RNA in qRT-PCR. The relative abundance of m^6^A RNA in the qRT-PCR amplicons was estimated using this algorithm: RA = 100 × 2^-ΔC^. Expected abundance (EA) of m^6^A RNA in the m^6^A-seq data set was estimated by this algorithm: EA = 100× (the mapped m^6^A RNA reads of the test gene in m^6^A-seq and in the cDNA region for qRT-PCR test/the mapped RNA reads of the *Actin2* gene in RNA-seq and in the cDNA region for qRT-PCR test). Consistency between the AR and ER results among three organs was compared (S2 Fig). The correlation coefficient between the average RA and the average EA was calculated using SPSS 13.0 (SPSS, USA).

## Results

### Extent of m^6^A methylation in the Arabidopsis chloroplast/amyloplast and mitochondria

**Fig 2:**
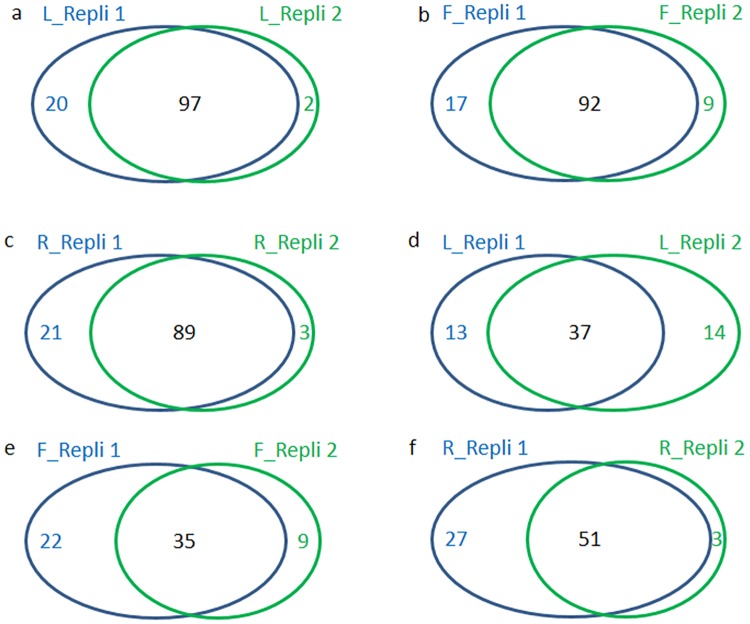
Number of the overlapped m^6^A transcripts in the two m^6^A-seq replicates. (a) in the leaf chloroplast; (b) in the flower chloroplast; (c) in the root amyloplast; (d)in the leaf mitochondria; (e) in the flower mitochondria; and (f) in the root mitochondria.

This study shared the same data sets with our recent publication for characterization of m^6^A methylation in the *Arabidopsis* nucleus [35]. Six samples from three organs of *Arabidopsis* leaves, flowers and roots were used for m^6^A-seq, six samples for RNA-seq, and six samples for input RNA-seq (total fragmented RNA without RIP experiment as the control for m^6^A-seq), respectively, with two replicates for each RNA sequencing (S2 Table). In m^6^A-seq, agreement proportion between two replicates was 82%, 78% and 79% from the leaf, flower and root chloroplast/amyloplast samples, respectively (Fig 2a–2c). While in the mitochondria, the agreement proportion was 74%, 70% and 77% from the leaf, flower and root samples, respectively (Fig 2d–2f).

In total, 133 and 146 genes have been so far discovered in the *Arabidopsis* chloroplast/amyloplast and mitochondria genome, respectively (Table 1)[36,44]. In this study, we found that 79–80% and 34–64% of the genes were transcribed in the chloroplast/amyloplast and mitochondria transcriptome, respectively (Table 1). This indicated that proportion of the transcribed genes in the chloroplast/amyloplast was close to that of the nuclear genes (*p*< 0.01)[35]. However, proportion of the transcribed genes in the mitochondria was significantly lower than that of the nuclear genes or the genes from the chloroplast/amyloplast (*p*< 10^−4^) (Table 1)[44].

**Table 1:** Proportion of the transcribed genes methylated by m^6^A in the chloroplast/amyloplast and mitochondria.

Transcribed genes	Chloroplast/ amyloplast			Mitochondria		
	Leaf	Flower	Root	Leaf	Flower	Root
Replicate 1						
Total number of the genes	133	133	133	146	146	146
Number of the transcribed genes	117	109	114	62	63	94
The transcribed genes methylated by m^6^A	117	109	110	51	57	78
Proportion of the transcribed genes (%)	88	82	86	42	43	64
Proportion of the methylated genes (%)	100	100	96	82	90	83
Replicate 2						
Number of the transcribed genes	99	101	92	56	49	56
The transcribed genes methylated by m^6^A	99	101	92	51	44	54
Proportion of the transcribed genes (%)	74	76	72	38	34	38
Proportion of the methylated genes (%)	100	100	100	91	90	96

We found that98–100%and 86–90% of the transcribed genes were chemically modified by m^6^A in the chloroplast/amyloplast and mitochondria transcriptome, respectively (Table 1). These two proportions were significantly higher than that (~73%) in the nuclear transcriptome (*p*< 10^−4^)[35]. The results also indicated that proportion of the m^6^A methylated genes in the chloroplast/amyloplast was higher than that in the mitochondria (*p*< 0.001) (Table 1).

On average, around 620 m^6^A sites from the leaves, ~580 sites from the flowers, and ~570 sites from the roots were successfully mapped to the *Arabidopsis* chloroplast genome with an estimation of ~5.6 to ~5.8 m^6^A sites per transcript (S3 Table). About 280 m^6^A sites from the leaves, ~340 sites from the flowers, and ~400 sites from the roots were successfully mapped to the *Arabidopsis* mitochondria genome with an estimation of ~4.6 to ~4.9 m^6^A sites per transcript (S4 Table). Therefore, the total m^6^A sites and the number of m^6^A sites per transcript in the chloroplast transcriptome were significantly higher than those in the mitochondria transcriptome(*p*< 0.01). In addition, the number of m^6^A sites per transcript in the two organelles was greatly higher than that in the nuclear transcritptome[33,35].

### m^6^A topology in the Arabidopsis chloroplast/amyloplast and mitochondria

**Fig 3:**
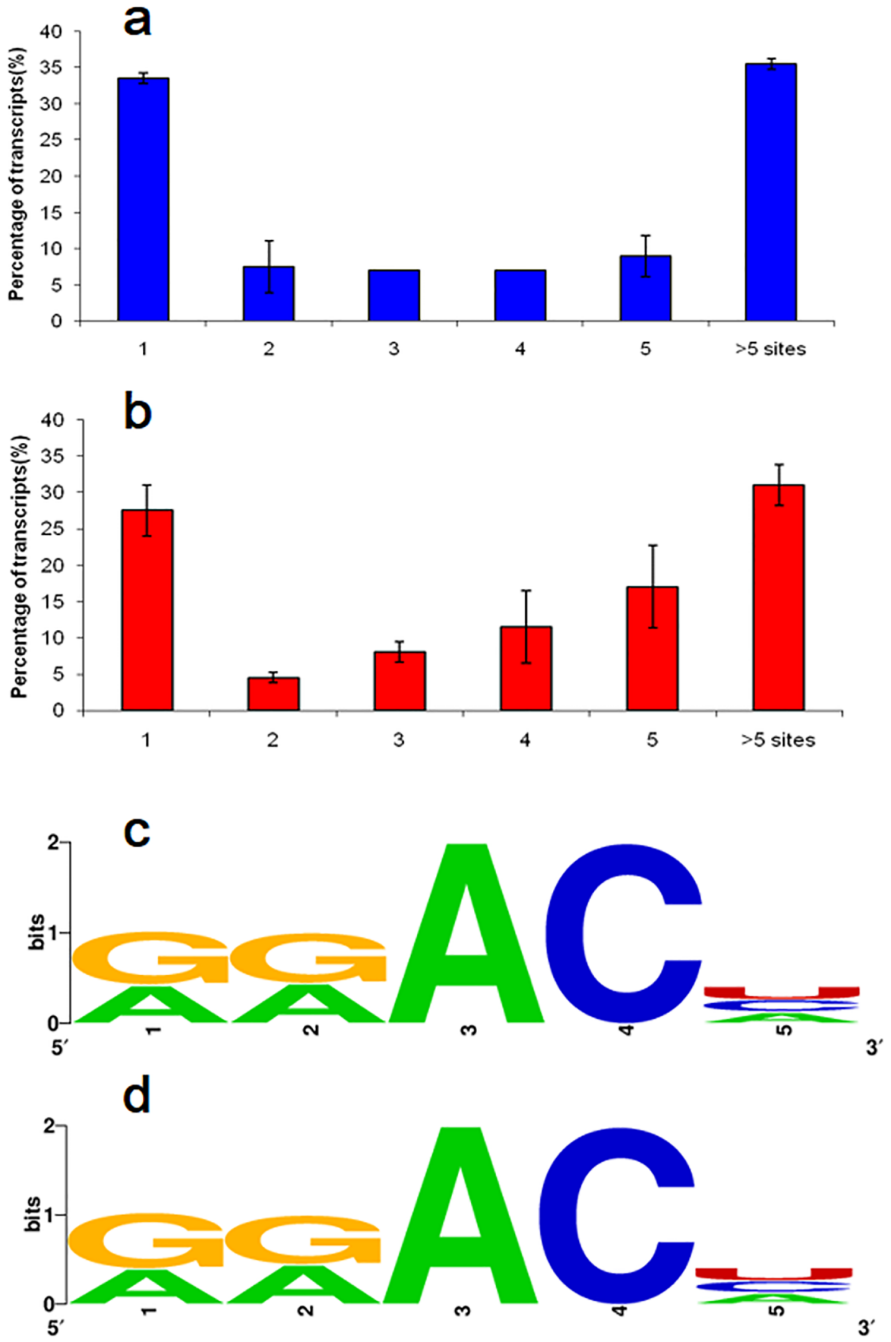
Features of m^6^A methylation in two organelles. (a) Proportion of the transcribed methylated genes containing different m^6^A sites in the chloroplast/amyloplast transcriptome; (b) proportion of the transcribed methylated genes containing different m^6^A sites in the mitochondria transcriptome; (c) the most common consensus motif (RRm^6^ACH) in the m^6^A peaks in the chloroplast/amyloplast transcriptome; and (d) the most common consensus motif (RRm^6^ACH) in the m^6^A peaks the mitochondria transcriptome.

Over 27% of the methylated transcripts was covered by one m^6^A site in the two organelles (Fig 3a and 3b, details in S5 and S6 Tables). Over 31% contained six or more sites in the two organelles (Fig 3a and 3b, details in S5 and S6 Tables).

The consensus sequence of m^6^A modification has been identified as ‘RRm^6^ACH’ in the nuclear transcritptome of mammals and plants, where R is A/G and H is A/C/U[17,21,35, 45]. Our data showed that over 65% and 67% of the RIP fragments in m^6^A-seq contained the consensus sequence of ‘RRm^6^ACH’ in the *Arabidopsis* chloroplast and mitochondria, respectively (Fig 3c and 3d). Two mostly observed motifs were GGm^6^ACC (10.3%) and GGm^6^ACU (10.7%) in the chloroplast transcriptome(Fig 3c). And GGm^6^ACA (10.4%) and GGm^6^ACU (11.0%) were the mostly detected motifs in the mitochondria transcriptome(Fig 3d). Thus the m^6^A motifs were conserved between the chloroplast and mitochondria transcriptome. Our observation also suggested that the m^6^A motifs in the two organelles were similar to those in the plant nuclear transcritptome[33,35].

**Fig 4:**
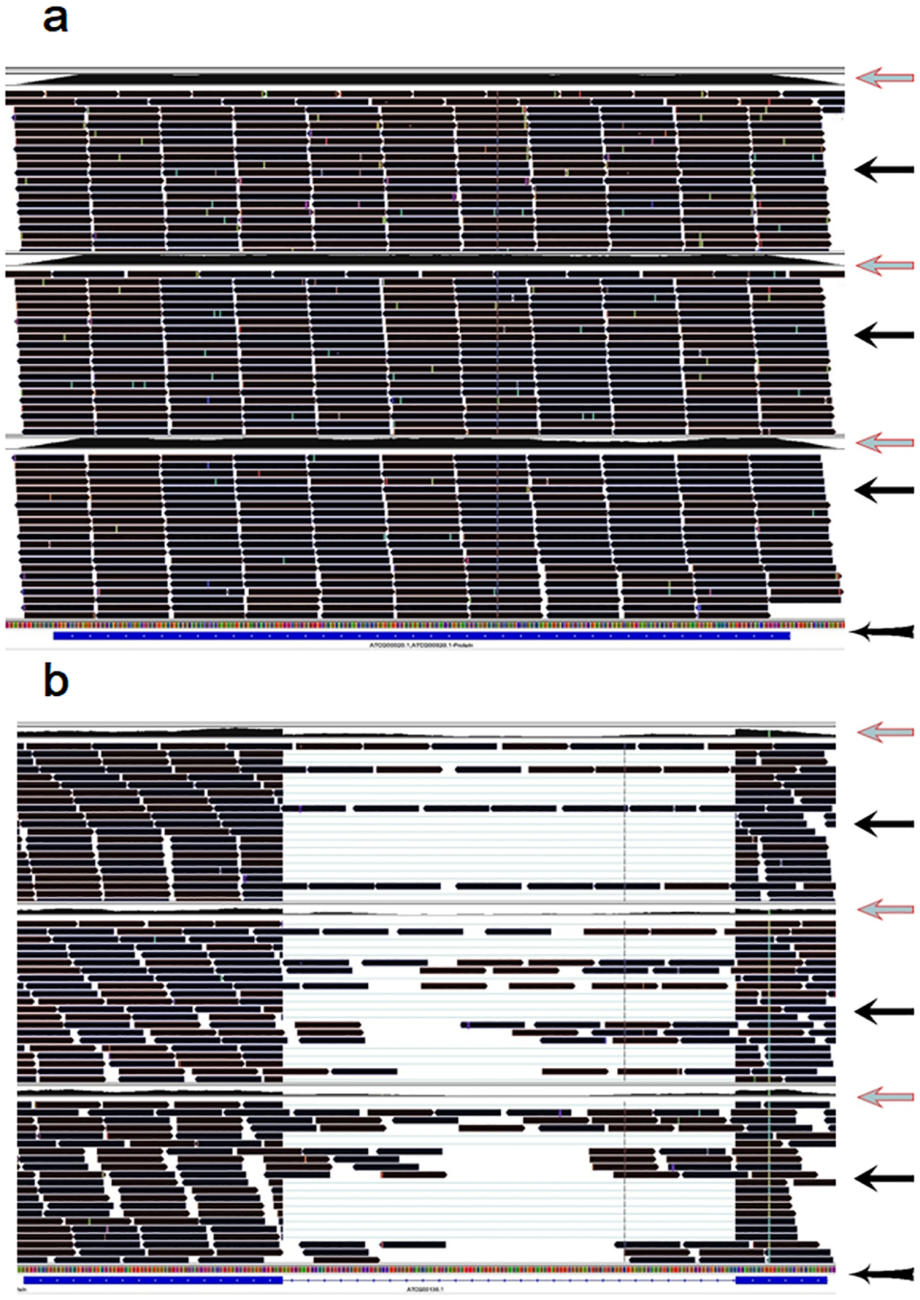
Screenshots from the IGV visualized program present two typical types of m^6^A topologies in the coding RNAs in the *Arabidopsis* chloroplast/amyloplast. Extent of m^6^A methylation, sequencing depth, sequencing fragment alignment, and gene ID of the sequencing data can be clearly visualized by the IGV program [42]. The area in the screenshot indicated by the arrow, “Red leftwards arrow”, presents m^6^A methylation extent across the transcript. The area in the screenshot indicated by the arrow, “Black leftwards arrow”, presents sequencing fragment alignment across the transcript. The area in the screenshot indicated by the arrow, “Black leftwards arrow with tail”, presents gene ID information including gene ID, sequence reading direction, the intron and exon regions. (a) Type 1 (representative gene, ‘ATCG00020’, expressed for ‘photosystem II reaction center protein A’), the whole transcript without intron was highly methylated by m^6^A; and (b) Type 2 (representative gene, ‘ATCG00130’, expressed for ‘ATPase, F0 complex, subunit B/B’), the exon was highly methylated but the intron was less methylated by m^6^A. Trace files of three organs, leaves (the upper), flowers (in the middle) and roots (the lower) were presented within a screenshot.

**Fig 5:**
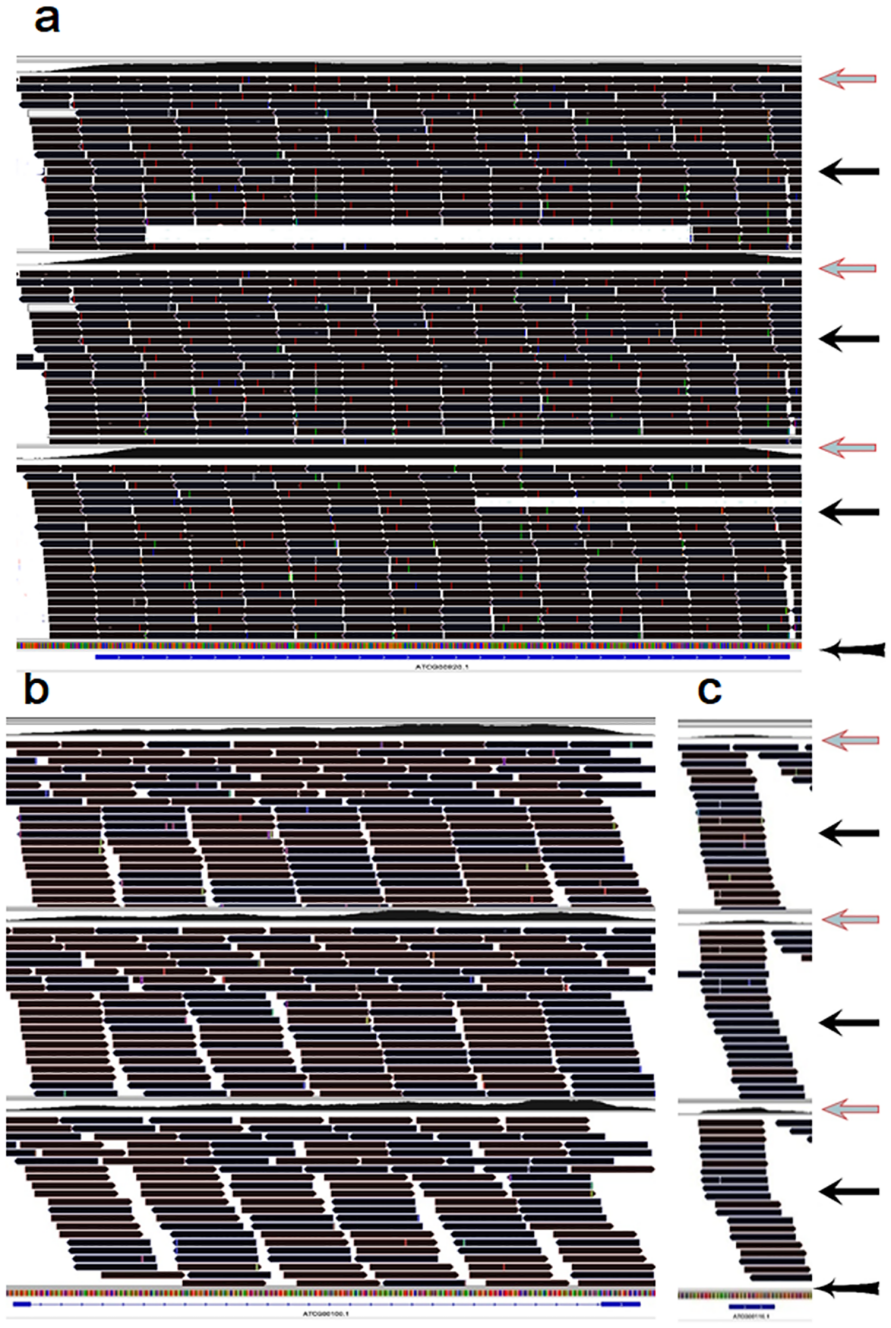
Screenshots from the IGV visualized program present m^6^A topologies in rRNA and tRNAs in the *Arabidopsis* chloroplast/amyloplast. Extent of m^6^A methylation, sequencing depth, sequencing fragment alignment, and gene ID of the sequencing data can be clearly visualized by the IGV program [42]. The area in the screenshot indicated by the arrow, “Red leftwards arrow”, presents m^6^A methylation extent across the transcript. The area in the screenshot indicated by the arrow, “Black leftwards arrow”, presents sequencing fragment alignment across the transcript. The area in the screenshot indicated by the arrow, “Black leftwards arrow with tail”, presents gene ID information including gene ID, sequence reading direction, the intron and exon regions. (a) The whole rRNA was highly methylated by m^6^A, representative rRNA, ‘ATCG00920’; (b) The whole tRNA with intron was highly methylated, representative tRNA, ‘ATCG00100’; and (c) The whole tRNA without intron was highly methylated, representative tRNA, ‘ATCG00110’. The Trace files of three organs, leaves (the upper), flowers (in the middle) and roots (the lower) were presented within a screenshot.

**Fig 6:**
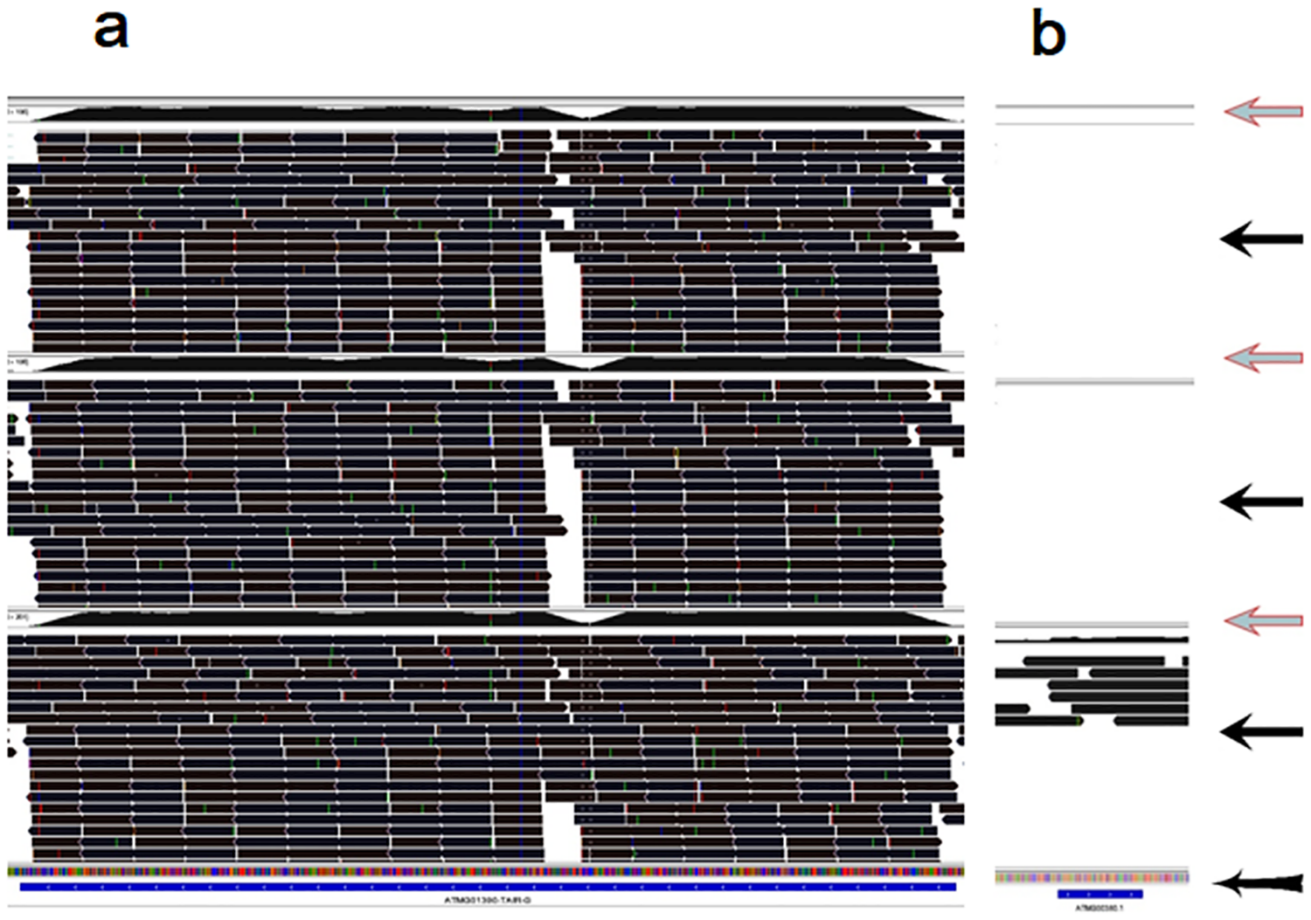
Screenshots from the IGV visualized program present m^6^A topologies in rRNA and tRNAs in the *Arabidopsis* mitochondria. Extent of m^6^A methylation, sequencing depth, sequencing fragment alignment, and gene ID of the sequencing data can be clearly visualized by the IGV program [42]. The area in the screenshot indicated by the arrow, “Red leftwards arrow”, presents m^6^A methylation extent across the transcript. The area in the screenshot indicated by the arrow, “Black leftwards arrow”, presents sequencing fragment alignment across the transcript. The area in the screenshot indicated by the arrow, “Black leftwards arrow with tail”, presents gene ID information including gene ID, sequence reading direction, the intron and exon regions. (a) The whole rRNA was highly methylated by m^6^A, representative rRNA, ‘ATMG01390’; (b) The whole tRNA was slightly methylated by m^6^A, representative tRNA, ‘ATMG00380’, expressed for tRNA-Asn. The Trace files of three organs, leaves (the upper), flowers (in the middle) and roots (the lower) were presented within a screenshot.

Most of the modified transcripts had similar m^6^A patterns between the two organelles (Figs 1 and 4–6). Two typical m^6^A patterns were found in the coding RNAs in the two organelles (Figs 1 and 4): (1) the whole coding RNA without intron was highly methylated by m^6^A (Figs 1a and 4a), and (2) the exon of the transcript was highly methylated but the intron was much less methylated by m^6^A (Figs 1b and 4b). All rRNAs were extensively methylated by m^6^A in the two organelles (Figs 5a and 6a). Most of tRNAs, with or without intron, were highly methylated by m^6^A in the *Arabidopsis* chloroplast/amyloplast (Fig 5b and 5c).

### m^6^A methylation extent versus gene transcript level in the two organelles

To compare m^6^A methylation extent in m^6^A-seq with gene transcript level in RNA-seq in *Arabidopsis*, our previous study categorized the m^6^A methylation extent into three groupings based on comparison of ‘the modified fragments per kilobase of transcript per million fragments mapped of the transcript in m^6^A-seq (MFPKM)’ with ‘the fragments per kilobase of transcript per million fragments mapped of the counterpart in the RNA-seq (FPKM)’using χ^2^ test[35]. This study also applied this method for analysis of extent of m^6^A methylation in the transcriptome of two organelles.

The chloroplast/amyloplast showed a different m^6^A methylation extent among three organs. On average, 79% and 52% of the methylated transcripts in the leaf and flower chloroplast showed a high m^6^A modification level, while 5% and 15% of the m^6^A modified transcripts had a low modification extent, respectively(Table 2). However, 33% of the methylated transcripts in the root amyloplast showed a high m^6^A modification level, and 40% had a low m^6^A modification level (Table 2).

**Table 2:** Three groupings of the m^6^A methlylation extent compared to the transcript level in the chloroplast/amyloplast transcriptome of three organs in *Arabidopsis*.

Plant organs	High		Low		Equivalent	
	No. of transcribed genes	Proportion(%)	No. of transcribed genes	Proportion(%)	No. of transcribed genes	Proportion(%)
Leaves_1	95	82	7	6	15	13
Leaves_2	75	76	4	4	20	19
Average		79		5		16
Flowers_1	54	50	26	24	29	26
Flowers_2	54	53	5	5	42	42
Average		52		15		34
Roots_1	47	43	52	47	11	10
Roots_2	21	23	29	32	42	45
Average		33		40		28

‘High’, ‘Low’ and ‘equivalent’ were categorized by comparison of the m^6^A-seq depth (MFPKM, the methlylation extent of m^6^A) of each transcript with that in the RNA-seq (FPKM, the transcript level). ‘High’ or ‘Low’ referred to as a relatively high or low m^6^A methlylation extent compared with its transcript level based on χ^2^ test (*p*< 0.05); ‘equivalent’, suggested that the m^6^A methlylation depth was relatively ‘equivalent’ to the transcript level (ratio of MFPKM to FPKM fits 1:1) based on χ^2^ test (*p*< 0.05).‘_1’ and ‘_2’ represent two replicates.

The mitochondria showed a similar m^6^A methylation trend in three organs of the leaf, flower and root. Proportion of three groupings representing m^6^A methylation level in the mitochondria had non-significant differences among three organs (*p*> 0.6) (Table 3). On average, 85–89% of the methylated transcripts had a high modification level, while 4–6% of the modified transcripts presented a low modification extent in the three organs. In addition, proportion of the transcripts showing a high methylation extent in the mitochondria was higher than that in the chloroplast/amyloplast (Tables 2 and 3).

**Table 3:** Three groupings of the m^6^A methlylation extent compared to the transcript level in the mitochondria transcriptome of three organs in *Arabidopsis*.

Plant organs	High		Low		Equivalent	
	No. of transcribed genes	Proportion(%)	No. of transcribed genes	Proportion(%)	No. of transcribed genes	Proportion(%)
Leaves_1	40	80	4	8	6	12
Leaves_2	46	90	2	4	3	6
Average		85		6		9
Flowers_1	52	91	2	4	3	5
Flowers_2	38	86	2	5	4	9
Average		89		4		7
Roots_1	69	89	5	6	4	5
Roots_2	48	89	2	4	4	7
Average		89		5		6

‘High’, ‘Low’ and ‘equivalent’ were categorized by comparison of the m^6^A-seq depth (MFPKM, the methlylation extent of m^6^A) of each transcript with that in the RNA-seq (FPKM, the transcript level). ‘High’ or ‘Low’ referred to as a relatively high or low m^6^A methlylation extent compared with its transcript level based on χ^2^ test (*p*< 0.05); ‘equivalent’, suggested that the m^6^A methlylation depth was relatively ‘equivalent’ to the transcript level (ratio of MFPKM to FPKM fits 1:1) based on χ^2^ test (*p*< 0.05).‘_1’ and ‘_2’ represent two replicates.

To better understand relationship between the m^6^A methylation extent in m^6^A-seq and the transcript level in RNA-seq in the chloroplast/amyloplast and mitochondria, the transcript level was categorized into three groupings: high, moderate and low as the method we previously used[35]. And each category contained one-third of the m^6^A modified transcripts from the highest to the lowest FPKM in RNA-seq as we described in our previous study[35].

In the both leaf and flower chloroplasts, comparison of ratio of average MFPKM in m^6^A-seq to average FPKM in RNA-seq between three groupings using *t*-test (Table 4) showed that most of the highly expressed transcripts were relatively less modified by m^6^A, and most transcripts with a low expression level were more likely modified by m^6^A (*p*<0.05). The moderately expressed transcripts tended to be moderately methylated in the leaf and flower chloroplasts (*p*< 0.05). However, the root amyloplast presented this methylation feature that the moderately expressed transcripts were more likely to be methylated, and those expressed at the two extremes were less methylated by m^6^A (Table 4). Intriguingly, the mitochondria transcripts in all three organs presented this feature: most of the highly expressed transcripts were relatively less methylated by m^6^A, and most transcripts with a low expression level were more likely modified by m^6^A (*p*< 0.05). The moderately expressed transcripts tended to be moderately methylated by m^6^A in the mitochondria (*p*< 0.05) (Table 5). Therefore, in most cases, features of the methylation extent versus the transcript level in the two organelles were similar to those recently found in the nuclear transcripts in *Arabidopsis*[35].

**Table 4:** Relationship between the m^6^A methlylation extent and the transcript level in the chloroplast/amyloplast transcriptome.

Plant organs	High			Moderate			Low		
	MFPKM	FPKM	Ratio	MFPKM	FPKM	Ratio	MFPKM	FPKM	Ratio
Leaves_1	82964.4	32491.2	2.6	6002.6	1990.8	3.0	558.6	230.3	2.4
Leaves_2	32128.9	33644.2	1.0	12436.3	5137.6	2.4	5792.8	1060.7	5.5
Average			1.8			2.7			4.0
Flowers_1	151788.5	53586.9	2.8	45698.8	6592.2	6.9	6448.6	896.4	7.2
Flowers_2	30304.4	28351.9	1.1	12931.0	7610.2	1.7	7183.2	2068.3	3.5
Average			1.9			4.3			5.4
Roots_1	113745.4	37647.1	3.0	36667.2	7074.2	5.2	4762.5	1708.5	2.8
Roots_2	26555.5	31256.0	0.8	14101.1	14295.3	1.0	10214.8	5962.2	1.7
Average			1.9			3.1			2.3

‘High’, ‘Moderate’, and ‘Low’ refers to as three groupings of the transcript levels from the highest to the lowest FPKM in RNA-seq. Each grouping included one-third numbers of the m^6^A modified transcripts. *t*-test on ratio of the average MFPKM in m^6^A-seq to the average FPKM in RNA-seq in each grouping showed significantly different (*p*< 0.05) ratios between three groupings. ‘_1’ and ‘_2’ represent two replicates.

**Table 5:** Relationship between the m^6^A methlylation extent and the transcript level in the mitochondria transcriptome.

Plant organs	High			Moderate			Low		
	MFPKM	FPKM	Ratio	MFPKM	FPKM	Ratio	MFPKM	FPKM	Ratio
Leaves_1	23180.3	22058.9	1.1	9544.0	2080.8	4.6	4633.4	403.9	11.5
Leaves_2	45144.4	39388.0	1.1	3678.0	1657.7	2.2	4282.0	373.6	11.5
Average			1.1			3.4			11.5
Flowers_1	39204.6	41593.0	1.0	6451.7	1122.6	5.7	9909.9	291.0	34.1
Flowers_2	44625.7	32068.5	1.4	4087.3	719.3	5.7	2412.2	178.6	13.5
Average			1.2			5.7			23.9
Roots_1	186592.6	25723.2	7.3	7697.9	587.1	13.1	4160.2	138.3	30.1
Roots_2	41081.8	38538.0	1.1	6729.9	667.1	10.1	1869.4	117.9	15.9
Average			4.4			12.6			23.0

‘High’, ‘Moderate’, and ‘Low’ refers to as three groupings of the transcript levels from the highest to the lowest FPKM in RNA-seq. Each grouping included one-third numbers of the m^6^A modified transcripts. *t*-test on ratio of the average MFPKM in m^6^A-seq to the average FPKM in RNA-seq in each grouping showed significantly different (*p*< 0.05) ratios between three groupings. ‘_1’ and ‘_2’ represent two replicates.

### The transcripts extensively methylated by m^6^A in organelles

We found that ~15%, 6% and 8% of the modified transcripts were extensively methylated by m^6^A in the chloroplast/amyloplast of leaves, flowers and roots, respectively (with a ratio of MFPKM in the m^6^A-seq to FPKM in the RNA-seq ≥ 5, False discovery rate (FDR)<10^−12^, and the cleaned read number per transcript ≥ 20) (Tables 6 and 7). In total, 20 transcripts were found extensively methylated by m^6^A in the chloroplast/amyloplast of the three *Arabidopsis* organs (Table 6). These transcripts extensively modified by m^6^A were mainly associated with chloroplast-encoded ribosomal RNA, ribosomal proteins, photosystem reaction proteins or tRNA (Table 6).

**Table 6:** The transcripts presenting extensive high m^6^A methylation in the *Arabidopsis* chloroplast/amyloplast.

Organs	Gene ID	Gene functions
Leaves	ATCG01160, ATCG00970, ATCG01210, ATCG01180, ATCG00950, ATCG00920	Chloroplast-encoded ribosomal RNA[54]
	ATCG00640, ATCG00650	ribosomal proteins[55,56]
	ATCG00400	tRNA[44]
	ATCG00550, ATCG00510	photosystem reaction proteins[57]
	ATCG00140	ATP synthase subunit C family protein[58]
	ATCG01130	Ycf1 protein[59]
	ATCG01010	NADH-Ubiquinone oxidoreductase[57]
	ATCG01040	Cytochrome C assembly protein[57]
	ATCG00660	PETG[55–57]
Flowers	ATCG01180, ATCG01210, ATCG00950, ATCG00920	Chloroplast-encoded ribosomal RNA[54]
	ATCG00550	photosystem reaction proteins[57]
	ATCG00140	ATP synthase subunit C family protein[58]
Roots	ATCG01180, ATCG00950, ATCG01210, ATCG00920	Chloroplast-encoded ribosomal RNA[60]
	ATCG01310, ATCG00110	ribosomal proteins[57,60]
	ATCG00390	tRNA[44]
	ATCG00890	NADH-Ubiquinone/plastoquinone (complex I) protein

**Table 7:** The transcripts presenting extensive high m^6^A methylation in the *Arabidopsis* mitochondria.

Organs	Gene ID	Gene functions
Leaves	ATMG01380, ATMG00020, ATMG01390	mitochondrial ribosomal RNA[54]
	ATMG00030, ATMG01200, ATMG01130	Proteins of unknown functions
	ATMG00650, ATMG00285	NADH dehydrogenase subunits[61]
	ATMG00160	Cytochrome oxidase 2
	ATMG00280	Ribulose bisphosphate carboxylase large chain
Flowers	ATMG01380, ATMG00020, ATMG01390	mitochondrial ribosomal RNA
	ATMG00510, ATMG00650, ATMG00060, ATMG00070, ATMG00580	NADH dehydrogenase subunits
	ATMG00640	hydrogen ion transporting ATP synthases
	ATMG01130, ATMG00030, ATMG00660, ATMG00690	Proteins of unknown functions
	ATMG00730	cytochrome c oxidase subunit
	ATMG00080	ribosomal protein
	ATMG01360	cytochrome oxidase
	ATMG01170	ATPase
Roots	ATMG01380	mitochondrial ribosomal RNA
	ATMG00285, ATMG00510, ATMG00580, ATMG00513, ATMG01120, ATMG01320, ATMG00270, ATMG00650	NADH dehydrogenase subunits
	ATMG00900, ATMG00830, ATMG00516, ATMG00180, ATMG00960	cytochrome C biogenesis
	ATMG00980, ATMG00210, ATMG00080	Ribosomal proteins
	ATMG00570	Sec-independent periplasmic protein translocase
	ATMG00220	apocytochrome b
	ATMG01190, ATMG00640	ATP synthase
	ATMG00060, ATMG01020, ATMG01130, ATMG00660, ATMG01320	Proteins of unknown functions
	ATMG00590	Cytochrome b/b6 protein
	ATMG00560	Nucleic acid-binding, OB-fold-like protein
	ATMG00640	hydrogen ion transporting ATP synthases
	ATMG01170	ATPase

And ~20%, 34% and 45% of the modified transcripts were extensively methylated by m^6^A in the mitochondria of leaves, flowers and roots, respectively. In total, 38 transcripts were discovered extensively methylated by m^6^A in the mitochondria of the three *Arabidopsis* organs (Table 7). These transcripts extensively modified by m^6^A were mainly related with mitochondria-encoded ribosomal RNA, ribosomal proteins, NADH dehydrogenase subunits, protein for redox, or proteins of unknown functions (Table 7).

### Differential m^6^A methylation across organs in the transcriptomes of two organelles

As we described in our previous study[35], we applied an algorithm ‘MFPKM in m^6^A-seq divided by LOG (FPKM in RNA-seq, 2) (NFPKM)’to each transcript to estimate differential m^6^A methylation among three organs of leaves, flowers and roots (see details in the Methods section of this paper). Two fold change and chi-square were applied for estimation of differential m^6^A methylation and differential gene transcript level between two organs[35].

On average, 72% of the transcripts in the chloroplast/amyloplast presented differential transcript level between two organs(fold change of FPKM between two organs > 2 or < 0.5, and FDR< 0.05). However, 64%of the modified transcripts in the chloroplast/amyloplast showed differential methylation between two organs (fold change of NFPKM between two organs > 2 or < 0.5, and FDR< 0.05) (Table 8). A paired analysis indicated that proportion of transcripts in the chloroplast/amyloplast showing differential transcript level across organs was higher than that showing differential m^6^A methylation extent among the three *Arabidopsis* organs (*p*< 0.003). On average, 69% of the transcripts in the mitochondria presented differential transcript level (fold change of FPKM between two organs > 2 or < 0.5, and FDR< 0.05). However, 79%of the m^6^A transcripts in the mitochondria showed differential methylation between two organs (fold change of NFPKM between two organs > 2 or < 0.5, and FDR< 0.05) (Table 9). Proportion of the transcripts in the mitochondria showing differential transcript level was significantly lower than that showing the differential m^6^A methylation extent among the three *Arabidopsis* organs (*p*< 0.05). The comparison also showed that two organelles in the leaves exhibited the highest extent of m^6^A methylation among three organs followed by that in the flower organelles. And the transcripts in the root organelles were less likely methylated among three organs (Table 8).

**Table 8:** The transcripts presenting differential transcript level and differential m^6^A methylation in the chloroplast/amyloplast among three organs in *Arabidopsis* (fold change >2 or <0.5, FDR < 0.02).

Differential level	Leaves vs Flowers		Leaves vs Roots		Flowers vs Roots	
	Hi-leaves	Hi-flowers	Hi-leaves	Hi-roots	Hi-flowers	Hi-root
Differential transcript level						
Replicate 1						
No. of transcripts	71	13	20	71	28	39
Proportion (%)	62	11	17	60	25	35
Total (%)	73		77		60	
Replicate 2						
No. of transcripts	59	17	20	59	21	61
Proportion (%)	52	15	20	59	20	58
Total (%)	67		79		78	
Differential m^6^A extent						
Replicate 1						
No. of transcripts	60	13	47	41	45	21
Proportion (%)	52	11	39	34	40	19
Total (%)	63		73		59	
Replicate 2						
No. of transcripts	34	30	36	24	30	23
Proportion (%)	36	32	40	27	33	25
Total (%)	68		67		58	

**Table 9:** The transcripts presenting differential transcript level and differential m^6^A methylation in the mitochondria among three organs in *Arabidopsis* (fold change >2 or <0.5, FDR < 0.02).

Differential level	Leaves vs Flowers		Leaves vs Roots		Flowers vs Roots	
	Hi-leaves	Hi-flowers	Hi-leaves	Hi-roots	Hi-flowers	Hi-root
Differential transcript level						
Replicate 1						
No. of transcripts	29	8	32	8	15	15
Proportion (%)	57	16	53	13	29	29
Total (%)	73		66		58	
Replicate 2						
No. of transcripts	27	8	32	3	28	0
Proportion (%)	61	18	67	6	64	0
Total (%)	79		73		64	
Differential m^6^A extent						
Replicate 1						
No. of transcripts	42	1	41	0	24	16
Proportion (%)	93	2	89	0	44	29
Total (%)	95		89		73	
Replicate 2						
No. of transcripts	20	14	29	5	23	5
Proportion (%)	44	31	63	11	55	12
Total (%)	75		74		67	

Six genes were randomly chosen for validation of our analysis of m^6^A differential methylation in the two organelles(S1 Table). Because the amplicons of qRT-PCR cover a short span in the transcriptome, 50 to 150 bp[33], two flanks of the amplicon containing one m^6^A peak in IGV program and showing differential m^6^A methylation were chosen to design primers (S1 Table). The correlation coefficient between the qRT-PCR and the RIP-seq results was significant (*r* = 0.9294, *n* = 18 genes, and *p*< 10^−5^), indicating that our qRT-PCR data were consistent with the data estimated by m^6^A-seq and RNA-seq using the IGV program (S2 Fig).

## Discussion

### Similarities and differences of m^6^A methylation between nucleus and organelle transcriptomes

MFPKM in m^6^A-seq represents the methylation level of the transcripts[35]. The average m^6^A MFPKM in the two organelles(Tables 4 and 5) was extensively (more than a hundred times) higher than that in the nucleus[35]. In addition, proportion of the m^6^A modified transcripts (over 90%) in the two organelles (Table 1) was also significantly higher than that (~73%) in the nucleus in *Arabidopsis*[35]. Therefore, the overall m^6^A methylation extent in the two organelles was greatly higher than that in the nucleus.

m^6^A motifs were similar between the nucleus and organelle transcriptome (Fig 3c and 3d), suggesting that recognition of motif for m^6^A methylation was conserved between the nucleus and the two organelles. Because the genes responsible for RNA m^6^A methylation have not been detected in the organelles, the enzymes of these genes may be expressed from nucleus and transported to the organelles form^6^A methylation therein.

m^6^A patterns in rRNAs were also similar between the nucleus and the organelles. For example, the whole rRNA transcripts were highly methylated by m^6^A in the both nucleus and organelles (Figs 5a and 6a)[35]. m^6^A patterns in tRNAs in the chloroplast/amyloplast were also similar to those in the nuclear transcripts (Fig 5b and 5c)[35]. However, m^6^A patterns in the coding RNAs were apparently different between nucleus and organelle transcripts. A dominant m^6^A peak nears top codon or in the 3′UTR was observed in most of the nuclear mRNA[14,17,18,23,35]. However, this dominant m^6^A peak was not observed in the coding RNAs in the two organelles (Figs 1 and 4). While most of the coding RNAs were highly methylated with numerous m^6^A peaks evenly distributing in the transcript exons including stop codon or 3′UTR though the extensively lower m^6^A peaks were observed in the introns of the coding RNAs in the two organelles(Figs 1 and 4). This suggested that regulation of the m^6^A methylation patterns may be somewhat different between the nuclei and the organelles.

This study also demonstrated that both of the transcript level and the m^6^A methylation extent in the transcriptome of two organelles(Tables 8 and 9) showed a higher differential ratio than that in the nuclear transcritptome[35].

### Similarities and differences of m^6^A methylation between chloroplast/amyloplast and mitochondria

m^6^A patterns in the coding RNAs and rRNAs were similar between two organelles in *Arabidopsis*(Figs 1 and 4–6), suggesting an alike machinery for m^6^A methylation between the chloroplast/amyloplast and mitochondria. However, the m^6^A patterns in tRNAs were distinct between two organelles. For example, most of tRNAs were highly methylated by m^6^A in the chloroplast/amyloplast (Fig 5c). However, only few tRNAs was detected to be methylated by m^6^A in the mitochondria (Fig 6b). This may be due to too low transcript level of tRNAs for detection of their m^6^A methylation because very few tRNAs with an extremely low transcript level were also observed in the RNA-seq data in the mitochondria.

The average m^6^A MFPKM in the chloroplast/amyloplast (Table 4) was significantly higher than that in the mitochondria (Table 5) (*p*<0.001). In addition, proportion of the m^6^A modified transcripts (nearly 100%) in the chloroplast/amyloplast was also significantly higher than that (over 90%) in the mitochondria (Table 1, *p*< 0.05). Therefore, the overall m^6^A methylation extent in the chloroplast/amyloplast was higher than that in the mitochondria.

### Potential functions of m^6^A methylation in the transcriptome of two organelles

m^6^A methylation in the nucleus acts as a signal for transport of RNA from the nucleus to the cytoplasm[17,21]. The dysfunction of m^6^A methylation may result in a failure of RNA transport from the nucleus to the cytoplasm, or RNA degradation[21]. Nuclear mRNAs translated into proteins located in mitochondria or chloroplast were also found highly methylated by m^6^A in our previous study[35]. The overall m^6^A methylation extent in the two organelles was found in this study (Tables 1, 4 and 5) extensively higher than that in the nuclear transcripts[35]. However, the biological functions responsible for this phenomenon need further investigation.

In the both chloroplast and mitochondria, introns were much less methylated than exons in the coding RNAs (Figs 1b and 4b). This phenomenon was similar to that in the nuclear mRNAs[35], suggesting that m^6^A methylation in the two organelles may also be responsible for RNA splicing[17,35]. Mitochondria confer a role of regulation of cellular proliferation and differentiation[46]. m^6^A in the nuclear transcripts is also related to regulation of differentiation and fate of the stem cells[10,47]. However, effects of m^6^A methylation in the organelles on the cellular proliferation and differentiation need further investigation.

Expressions of some genes were mutually regulated by each other between the organelle and the nucleus. Whether and how m^6^A methylation in the nucleus regulates gene expression in the two organelles, or *vice versa*, is unclear. Gene silencing of *METTL3*, a gene responsible for m^6^A modification, can result in an arrest of embryo development at the globular stage in *Arabidopsis*[28]. The male-infertility line plays an important role in crop breeding[48–51]. Some infertility phenomena are caused by organelle dysfunctions or interactions between the organelle and nucleus genes[52,53]. Nevertheless, an investigation whether m^6^A methylation in the organelles affects fertility and development of the reproductive organ may provide insights in our future breeding programs.

High productivity in crops is highly related to relative high photosynthesis and low respiration in plants[36,37]. Chloroplast will be switched into amyloplast in mature seeds, fruits or tubes, and mainly used for food storage in plants[37]. The transcripts expressed for photosystem reaction proteins, NADH dehydrogenase subunits, and protein for redox, were extensively methylated by m^6^A in the two organelles (Tables 6 and 7). m^6^A methylation has been found to have function in regulation of gene expression[23,24]. Further studies in investigation of molecular functions of m^6^A methylation in the chloroplast/amyloplast and mitochondria may facilitate our better control of metabolisms in these two organelles, thus to potentially increase crop productivity to ensure the global food and energy security in the future.

## Conclusions

To our knowledge, this is the first study for comprehensive and transcriptome-wide characterization of RNA m^6^A patterns, relationship between m^6^A methylation extent and gene transcript level, and differential features of m^6^A methylation across leaves, flowers and roots in the chloroplast/amyloplast and mitochondria.

Over 600 and 400 m^6^A sites were successfully mapped to the *Arabidopsis* chloroplast/amyloplast and mitochondrial genomes, respectively, with an estimation of ~4.6 to ~5.8 m^6^A sites per m^6^A transcript, in the two organelles. Over 86% of the transcripts were chemically modified by m^6^A in the two organelle transcriptomes. Around two thirds of the m^6^A sites in the transcripts in the two organelles contained motifs, ‘RRm^6^ACH’, which were similar to that in the nuclear transcripts. The average MFPKM of m^6^A-seq in the chloroplast/amyloplast and mitochondria was over a hundred times higher than that in the nucleus. In most cases, the m^6^A methylation extent was relatively high compared to the transcript level in the two organelles (*p*< 0.05). The m^6^A extensively methylated transcripts in the two organelles were mainly associated with ribosomal RNA, ribosomal proteins, photosystem reaction proteins, NADH dehydrogenase subunits, protein for redo or tRNA. The m^6^A patterns in rRNAs were similar between the nucleus and organelle transcripts, i.e, the whole rRNAs were highly methylated by m^6^A. A dominant m^6^A peak enriched near stop codon or at 3'UTR in most of the nuclear mRNAs was not observed in the coding RNAs in the chloroplast/amyloplast and mitochondria. On average, 64% and 79% of the transcripts showed differential m^6^A methylation across three organs in the chloroplast/amyloplast and mitochondria, respectively. Intriguingly, the overall m^6^A methylation extent in the chloroplast/amyloplast was greatly higher than that in the mitochondria.

## Supporting information

S1 Fig RNA QC results of the total RNA and RNA for m^6^A-seq samples. (A) RNA quality of the total RNA for the RNA-seq sample was high with RIN over 8.5. (B) RNA fragmentation for the m^6^A-seq samples was consistent in the RIP experiments, with an average length of 106 nt.
(PDF)

S2 Fig The relative abundance (RA) of m^6^A RNA deduced from qRT-PCR and the expected abundance (EA) of m^6^A RNA deduced from the m^6^A-seq data set. (A) RA for ‘ATCG00360’. (B) EA for ‘ATCG00360’. (C) RA for ‘ATCG0083’. (D) EA for ‘ATCG0083’. (E) RA for ‘ATCG00890’. (F) EA for ‘ATCG00890’. (G) RA for ‘ATMG00510’. (H) EA for ‘ATMG00510’. (I) RA for ‘ATMG00513’. (J) RA for ‘ATMG00513’. (K) RA for ‘ATMG00580’. (L) EA for ‘ATMG00580’.
(PDF)

S1 Table The primers used for qRT-PCR. (PDF)

S2 Table The sequenced and mapped reads in the m^6^A-seq, mRNA-seq and input RNA-seq samples. (PDF)

S3 Table Number of m^6^A sites detected in the three organs of the *Arabidopsis* chloroplast/amyloplast. (PDF)

S4 Table Number of m^6^A sites detected in the three organs of the *Arabidopsis* mitochondria. (PDF)

S5 Table Category of the modified transcripts based on the number of m^6^A sites per transcript in the chloroplast/amyloplast. (PDF)

S6 Table Category of the modified transcripts based on the number of m^6^A sites per transcript in the mitochondria. (PDF)
