# Supplementary material for: High-throughput m6A-seq reveals RNA m6A methylation patterns in the chloroplast and mitochondria transcriptomes of Arabidopsis thaliana
Source: PLoS One. 2017 Nov 13;12(11):e0185612. doi: 10.1371/journal.pone.0185612 (PMC5683568; doi:10.1371/journal.pone.0185612)
Supplement: S1 Fig — (A) RNA quality of the total RNA for the RNA-seq sample was high with RIN over 8.5. (B) RNA fragmentation for the m6A-seq samples was consistent in the RIP experiments, with an average length of 106 nt. (PDF) [file pone.0185612.s001.pdf]

A For total RNA **B For RIP RNA sample**

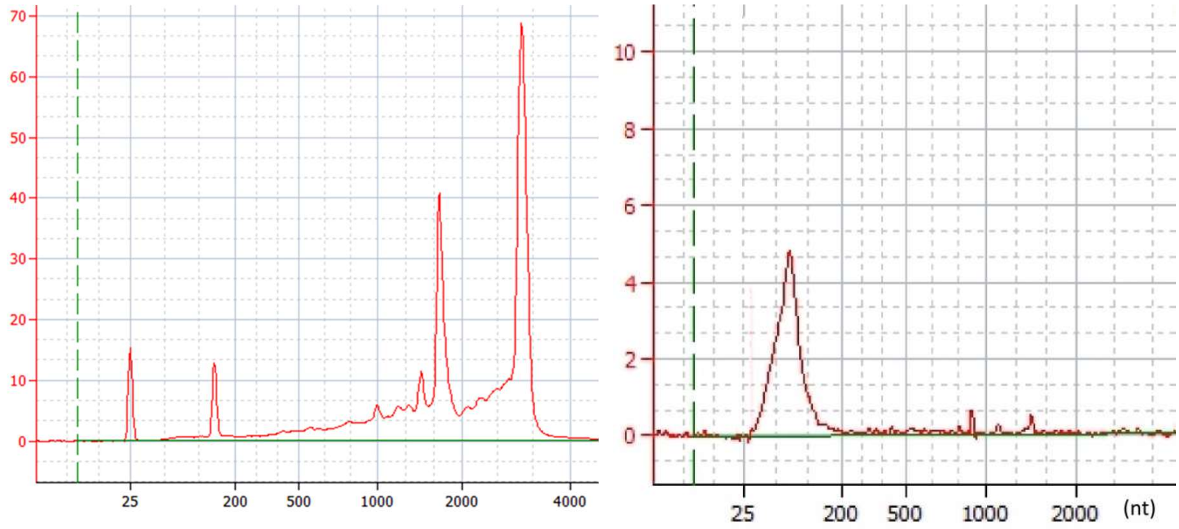

S1 Fig. RNA QC results of the total RNA and RNA for m^{6}A-seq samples.

(A) RNA quality of the total RNA for the RNA-seq sample was high with RIN over 8.5. (B) RNA fragmentation for the m6A-seq samples was consistent in the RIP experiments, with an average length of 106 nt.
