# Supplementary material for: High-throughput m6A-seq reveals RNA m6A methylation patterns in the chloroplast and mitochondria transcriptomes of Arabidopsis thaliana
Source: PLoS One. 2017 Nov 13;12(11):e0185612. doi: 10.1371/journal.pone.0185612 (PMC5683568; doi:10.1371/journal.pone.0185612)
Supplement: S2 Fig — (A) RA for ‘ATCG00360’. (B) EA for ‘ATCG00360’. (C) RA for ‘ATCG0083’. (D) EA for ‘ATCG0083’. (E) RA for ‘ATCG00890’. (F) EA for ‘ATCG00890’. (G) RA for ‘ATMG00510’. (H) EA for ‘ATMG00510’. (I) RA for ‘ATMG00513’. (J) RA for ‘ATMG00513’. (K) RA for ‘ATMG00580’. (L) EA for ‘ATMG00580’. (PDF) [file pone.0185612.s002.pdf]

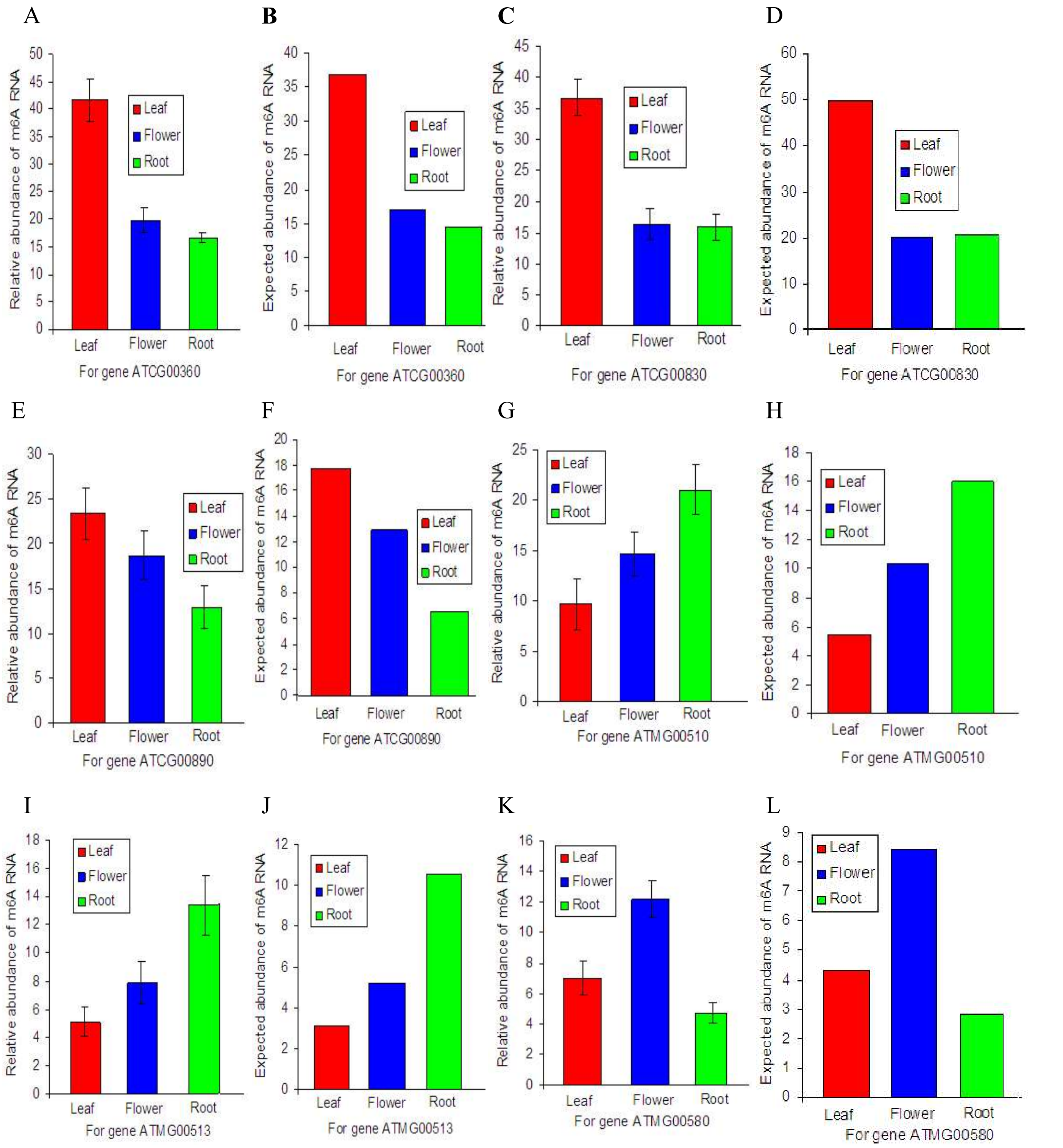

S2 Fig. The relative abundance (RA) of m^{6}A RNA deduced from qRT-PCR and the expected abundance (EA) of m^{6}A RNA deduced from the m^{6}A-seq data set.

(A) RA for 'ATCG00360'. (B) EA for 'ATCG00360'. (C) RA for 'ATCG0083'. (D) EA for 'ATCG0083'. (E) RA for 'ATCG00890'. (F) EA for 'ATCG00890'. (G) RA for 'ATMG00510'. (H) EA for 'ATMG00510'. (I) RA for 'ATMG00513'. (J) RA for 'ATMG00513'. (K) RA for 'ATMG00580'. (L) EA for 'ATMG00580'.
