# Supplementary material for: High-throughput m6A-seq reveals RNA m6A methylation patterns in the chloroplast and mitochondria transcriptomes of Arabidopsis thaliana
Source: PLoS One. 2017 Nov 13;12(11):e0185612. doi: 10.1371/journal.pone.0185612 (PMC5683568; doi:10.1371/journal.pone.0185612)
Supplement: S1 Table — (PDF) [file pone.0185612.s003.pdf]

S1 Table. The primers used for qRT-PCR

Gene ID	Forward primers	Reverse primers
ATCG00360	GGCATTAGAACGAAACCCCTT	TGGCCTGTTCTCCACGGTAA
ATCG00830	ACCGATATGCCCTTAGGCAC	TCAGTTTCGCTACAGCACCC
ATCG00890	GGTCTACGTACCGGAACTGAT	ACATAGAGCTAAAGAGAGAGCCA
ATMG00510	CACGATTAGTATTGGAAATGAACGG	TCTATTAATTTCTCAGTCCCTCTATGG
ATMG00513	GGCTCTCGGGAGTCTCTTTG	TACCGAGCAGGGGCAAAAAT
ATMG00580	TGTTTTTCCCGAAAGCGTGC	TCTTTCTTTGTCTCGAACCCCA
AT3G18780-*Actin2*	TCGCCATCCAAGCTGTTC	ACACCATCACCAGAATCCAG
