# Supplementary material for: High-throughput m6A-seq reveals RNA m6A methylation patterns in the chloroplast and mitochondria transcriptomes of Arabidopsis thaliana
Source: PLoS One. 2017 Nov 13;12(11):e0185612. doi: 10.1371/journal.pone.0185612 (PMC5683568; doi:10.1371/journal.pone.0185612)
Supplement: S2 Table — (PDF) [file pone.0185612.s004.pdf]

S2 Table. The sequenced and mapped reads in the m^{6}A-seq, mRNA-seq and input RNA-seq samples

Replicate	Samples	Tissues	Category	Clean reads after a removal of adaptors	Mapped reads	Mapped reads of the transcripts in chloroplast	Mapped reads of the transcripts in chloroplast
Replicate 1	zhwA01	Leaf	m^{6}A-seq	134,797,106	87,455,788	6,977,682	135,580
	zhwF15	Flower	m^{6}A-seq	115,165,660	33,489,846	411,880	124,614
	zhwRoot8	Root	m^{6}A-seq	104,774,124	75,518,793	530,572	313,220
	zhwLD24	Leaf	mRNA-seq	77,905,520	73,460,466	91,312	8,404
	zhwF15	Flower	mRNA-seq	60,349,042	57,139,889	11,534	13,404
	zhwRoot8CK	Root	mRNA-seq	92,491,920	87,205,832	19,640	125,820
	Leaf_rep1	Leaf	Input, RNA-seq	51,074,102	7,783,758		
	Flower_rep1	Flower	Input, RNA-seq	33,603,842	19,817,745		
	Root_rep1	Root	Input, RNA-seq	31,656,580	16,944,034		
Replicate 2	LE03	Leaf	m^{6}A-seq	90,726,318	70,600,665	70,522	22,792
	F23	Flower	m^{6}A-seq	165,894,986	125,665,375	54,370	25,258
	R07	Root	m^{6}A-seq	156,536,156	125,893,506	50,732	34,016
	L1	Leaf	mRNA-seq	59,673,962	53,807,285	44,644	13,572
	F1	Flower	mRNA-seq	48,964,626	40,562,265	22,644	23,830
	R1	Root	mRNA-seq	48,106,874	39,923,446	7,068	80,622
	Leaf_rep2	Leaf	Input, RNA-seq	53,643,026	9,208,929		
	Flower_rep2	Flower	Input, RNA-seq	25,613,866	16,322,980		
	Root_rep2	Root	Input, RNA-seq	30,648,326	16,678,032		
