# Supplementary material for: High-throughput m6A-seq reveals RNA m6A methylation patterns in the chloroplast and mitochondria transcriptomes of Arabidopsis thaliana
Source: PLoS One. 2017 Nov 13;12(11):e0185612. doi: 10.1371/journal.pone.0185612 (PMC5683568; doi:10.1371/journal.pone.0185612)
Supplement: S3 Table — (PDF) [file pone.0185612.s005.pdf]

S3 Table. Number of m^6A sites detected in the three organs of the *Arabidopsis* chloroplast/amyloplast

Replicates		Leaves	Flowers	Roots
Replicate 01	Number of the m^6A transcripts	117	109	110
	Total m^6A sites	700	637	652
	m^6A sites per m^6A transcript	6.0	5.8	5.9
Replicate 02	Number of the m^6A transcripts	98	100	92
	Total m^6A sites	544	525	503
	m^6A sites per m^6A transcript	5.6	5.3	5.5
