# Supplementary material for: High-throughput m6A-seq reveals RNA m6A methylation patterns in the chloroplast and mitochondria transcriptomes of Arabidopsis thaliana
Source: PLoS One. 2017 Nov 13;12(11):e0185612. doi: 10.1371/journal.pone.0185612 (PMC5683568; doi:10.1371/journal.pone.0185612)
Supplement: S4 Table — (PDF) [file pone.0185612.s006.pdf]

S4 Table. Number of m^6A sites detected in the three organs of the *Arabidopsis* mitochondria

Replicates		Leaves	Flowers	Roots
Replicate 01	Number of the m^6A transcripts	59	68	83
	Total m^6A sites	257	355	427
	m^6A sites per m^6A transcript	4.5	5.2	5.1
Replicate 02	Number of the m^6A transcripts	66	72	82
	Total m^6A sites	315	338	383
	m^6A sites per m^6A transcript	4.7	4.7	4.7
