# Supplementary material for: High-throughput m6A-seq reveals RNA m6A methylation patterns in the chloroplast and mitochondria transcriptomes of Arabidopsis thaliana
Source: PLoS One. 2017 Nov 13;12(11):e0185612. doi: 10.1371/journal.pone.0185612 (PMC5683568; doi:10.1371/journal.pone.0185612)
Supplement: S5 Table — (PDF) [file pone.0185612.s007.pdf]

S5 Table. Category of the modified transcripts based on the number of m^6A sites per transcript in the chloroplast/amyloplast

Replicates	Plant organs	Number of the transcripts with different m^6A sites (%)					
		1 site	2 sites	3 sites	4 sites	5 sites	> 5 sites
Replicate 1	Leaf	41	11	8	8	8	41
	flower	37	12	7	7	8	37
	root	37	12	9	7	6	39
	Average						
Replicate 2	Leaf	35	5	6	6	12	34
	flower	32	5	8	7	10	38
	root	30	5	7	7	11	32
	Average						
