# Supplementary material for: High-throughput m6A-seq reveals RNA m6A methylation patterns in the chloroplast and mitochondria transcriptomes of Arabidopsis thaliana
Source: PLoS One. 2017 Nov 13;12(11):e0185612. doi: 10.1371/journal.pone.0185612 (PMC5683568; doi:10.1371/journal.pone.0185612)
Supplement: S6 Table — (PDF) [file pone.0185612.s008.pdf]

S6 Table. Category of the modified transcripts based on the number of m^6A sites per transcript in the mitochondria

Replicates	Plant organs	Number of the transcripts with different m^6A sites (%)					
		1 site	2 sites	3 sites	4 sites	5 sites	> 5 sites
Replicate 1	Leaf	18	1	4	11	7	18
	flower	21	5	4	11	7	20
	root	24	6	7	9	15	23
	Average						
Replicate 2	Leaf	17	1	7	6	12	18
	flower	17	4	6	4	16	25
	root	19	4	6	7	16	26
	Average						
